# Research Progress of Green Solvent in CsPbBr_3_ Perovskite Solar Cells

**DOI:** 10.3390/nano13060991

**Published:** 2023-03-09

**Authors:** Jiajie Cheng, Zhenjun Fan, Jingjing Dong

**Affiliations:** School of Science, China University of Geosciences Beijing, No. 29 College Road, Haidian District, 100083 Beijing, China

**Keywords:** green solvent, CsPbBr_3_, toxic, perovskite solar cells

## Abstract

In optoelectronic applications, all-Brominated inorganic perovskite CsPbBr_3_ solar cells have received a great deal of attention because of their remarkable stability and simplicity of production. Most of the solvents used in CsPbBr_3_ perovskite solar cells are toxic, which primarily hinders the commercialization of the products. In this review, we introduce the crystal structure and fundamental properties of CsPbBr_3_ materials and the device structure of perovskite cells, summarize the research progress of green solvents for CsPbBr_3_ PSCs in recent years from mono-green solvent systems to all-green solvent systems, and discuss the approaches to improving the PCE of CsPbBr_3_ PSCs, intending to facilitate the sustainable development of CsPbBr_3_ perovskite solar cells. Finally, we survey the future of green solvents in the area of CsPbBr_3_ perovskite solar cells.

## 1. Introduction

Perovskite materials have unique and excellent photovoltaic properties, including high defect tolerance, adjustable band gap, and long exciton diffusion length [1,2]. Since perovskite materials were first used to prepare solar cells in 2009, their power conversion efficiency (PCE) has made continuous breakthroughs [3]. The highest PCE of perovskite solar cells (PSCs) has reached 25.7% [4]. In contrast to traditional silicon-based solar cells, new perovskite solar cells are easy to prepare and cost less. Due to these benefits, perovskite solar cells are becoming prominent. These advantages make perovskite solar cells a popular material for a new generation of solar cells [5].

Organic–inorganic hybrid perovskite solar cells have high power conversion efficiency due to their strong light absorption [6]. The maximum PCE of a single perovskite solar cell reached 25.7% [4]. Because organic components in organic–inorganic hybrid perovskite materials, such as CH_3_NH_3_^+^ (MA^+^) and CH(NH_2_)_2_^+^ (FA^+^), are highly volatile and degradative, a significant number of high-efficiency solar cells based on these materials experience instability under heat, humidity, and some other conditions [7]. To solve this instability issue, creating inorganic perovskite materials by completely substituting the organic cations with their inorganic counterparts is an attractive strategy [8,9,10]. 

All-inorganic Cesium Lead halide perovskites (CsPbX_3_, X = I, Br, Cl) have recently been the focus of extensive studies as light-absorbing materials due to their excellent stability and photoelectric characteristics [11,12,13]. The efficiency of all inorganic perovskite solar cells of the CsPbI_3_ system has achieved over 20% compared to conventional organic–inorganic hybrid perovskite solar cells [14,15,16]. The band gap of CsPbI_3_ is relatively narrow (1.73 eV) [16]. 

Therefore, achieving high power conversion efficiency is easy. Nevertheless, due to its low perovskite phase tolerance factor (0.8), the perovskite phase of CsPbI_3_ easily converts to the non-perovskite δ phase in a typical atmosphere [17]. Therefore, CsPbI_3_ perovskite solar cells cannot be prepared in a normal atmospheric environment. Improving the stability of CsPbI_3_ perovskite solar cells has become an important issue.

In order to solve this issue, researchers introduced Br to the CsPbI_3_ system to prepare CsPbIBr_2_, CsPbI_2_Br, and CsPbI_3−x_Br_x_ solar cells and to inhibit phase transformation [11,18]. The maximum PCE obtained among all Bromine-doped inorganic perovskite solar cells was 18.64% [19]. However, all inorganic perovskite solar cells doped with Bromine are still not stable, the majority of which need to be prepared in glove boxes.

Full-Brominated perovskite CsPbBr_3_ materials have gained much attention because of their excellent heat stability and moisture stability. It has been considered as a representative of all inorganic perovskite materials [20,21,22]. Compared to CsPbI_3_ or CsPbI_3-x_Br_x_ perovskite solar cells, CsPbBr_3_ PSCs can maintain an initial efficiency of more than 80% after exposure to air for 800 h [23]. Meanwhile, the band gap of CsPbBr_3_ is 2.3 eV [24], and the highest theoretical open-circuit voltage (Voc) for CsPbBr_3_ PSCs is 1.98 V [10], which makes CsPbBr_3_ perovskite material be utilized to fabricate stacked solar cells as a module. Therefore, the development of wide band gap CsPbBr_3_-based PSCs has broad application prospects. In general, for preparing high-performance CsPbBr_3_ PSCs, gaining a high-quality perovskite film with complete coverage and high crystallinity is crucial. Similar to the organic–inorganic hybrid perovskite films, the solution processing method [24,25] and the dual-source vacuum coevaporation (DSVE) method are widely used to build all-inorganic CsPbBr_3_ films [26,27].

**Figure 1:**
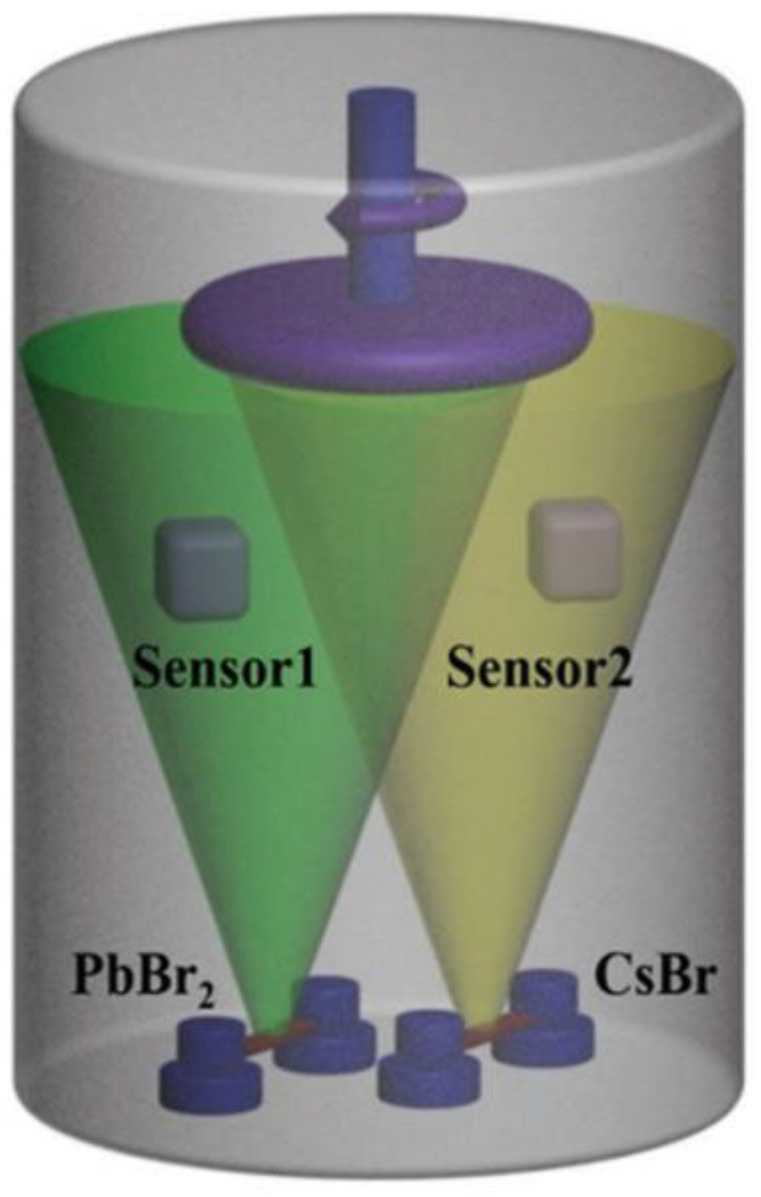
Schematic diagram of the DSVE method [26].

The dual-source vacuum coevaporation method uses vacuum evaporation equipment to heat the two reactants (PbBr_2_ and CsBr) on the evaporating dish so that two reactants can be deposited on the substrate [26]. The schematic diagram of the DSVE method is shown in Figure 1. This method can be used to control the deposition rate by controlling the substrate’s current and rotation rate. In addition, due to the relatively vacuum stable deposition environment, it is easy to deposit a large area of multilayer films with good uniformity and smoothness.

**Figure 2:**
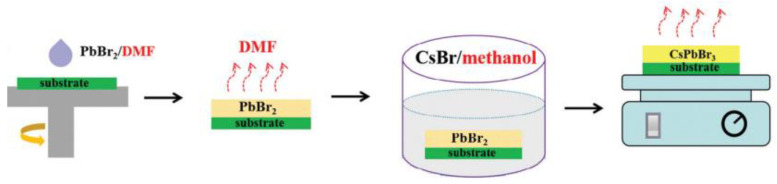
Schematic illustration of the preparation process of CsPbBr_3_ PSCs through a two-step soaking method [32].

When using the solution processing method, it is hard to form sufficiently uniform and thick CsPbBr_3_ films in a one-step solution method owing to the vastly different solubility of Lead Bromide (PbBr_2_) and Cesium Bromide (CsBr) in organic solvents [28]. To solve the solubility difference in the precursor solution, at present, the methods for preparing CsPbBr_3_ film by solution processing method mainly include a two-step soaking method and a multi-step spin-coating method [25,29,30,31]. Generally speaking, for the two-step soaking method depicted in Figure 2, the electron transport layer is initially spin-coated with PbBr_2_/DMF solution, and the deposited PbBr_2_ film is subsequently submerged in CsBr/methanol solution at a specific temperature. This soaking method is not conducive to controlling the degree of reaction, resulting in poor purity of CsPbBr_3_ crystal. 

**Figure 3:**
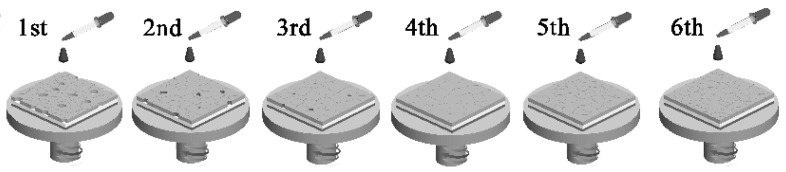
Schematic illustration diagram of multi-step spin-coating of CsBr solution [33].

The multi-step spin-coating method is based on the traditional two-step spin-coating method. It is challenging to prepare compact and pure CsPbBr_3_ film via the two-step spin-coating method because the solubility of CsBr in methanol is too low to form pure perovskite films. Tang’s group adopted a multi-step spin-coating method to prepare high-purity and high-quality CsPbBr_3_ perovskite films that can be used in effective solar cells. As depicted in Figure 3, by adjusting the cycle count for coating (*n*) of CsBr/methanol solution, the phase conversion from CsPb_2_Br_5_(*n* ≤ 3) to CsPbBr_3_(*n* = 4), and Cs_4_PbBr_6_(*n* > 4) was achieved [33].

Though CsPbBr_3_-based PSCs prepared with the multi-step spin-coating method have achieved PCE beyond 10% [34,35,36], the multi-step spin-coating method is quite laborious and it takes a while to complete the full conversion of PbBr_2_ and CsBr into CsPbBr_3_. Additionally, the most commonly used solvents for dissolving CsBr and PbBr_2_ in the multi-step spin-coating method are methanol and DMF or DMSO, which are highly toxic to humans. Finding substitutes for traditional solvents is necessary in order to achieve less hazardous physical and chemical reactions for human health, that are more eco-friendly while remaining equally effective. These alternative solvents are called green solvents [37].

Recently, researchers have started replacing extremely toxic anti-solvents with low-toxicity/non-toxic solvents to address the problem of highly toxic anti-solvents. The conventional hazardous anti-solvents have been replaced by a variety of non-toxic solvents, such as anisole [38,39], ethyl acetate [40], tetraethyl orthosilicate [41], and methyl benzoate [42]. However, research on the impact of green anti-solvent on inorganic CsPbBr_3_ PSCs is irrelevant, because inorganic CsPbBr_3_ perovskite solar cells are typically prepared with the two- or multi-step spin-coating method. Conversely, it is of great significance to study the influence of green solvents on CsPbBr_3_ PSCs because it can facilitate the progress of civilization in an ecologically friendly manner, and protect the health of researchers.

In this review, we briefly introduce the crystal structure and fundamental properties of CsPbBr_3_ materials and the device structure of perovskite cells, summarize the research progress of green solvents for CsPbBr_3_ PSCs in recent years from mono-green solvent systems to all-green solvent systems, and then discuss the approaches to improving the PCE of CsPbBr_3_ PSCs. Finally, the prospect of green solvents in the domain of CsPbBr_3_ PSCs is also discussed.

## 2. Crystal Structure and Basic Properties of CsPbBr_3_

**Figure 4:**
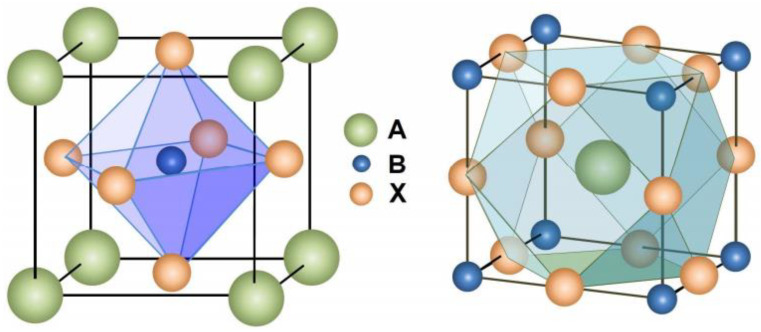
The basic structure of perovskite ABX3 [43].

The structural formula of perovskite material is ABX_3_ [43], and its basic structure is shown in Figure 4, where A represents monovalent cations (Cs^+^, MA^+^, or FA^+^), B represents divalent cations (Pb^2+^, Sn^2+^, or Ge^2+^), and X represents halogen anions (Cl^−^, Br^−^, or I^−^).

**Figure 5:**
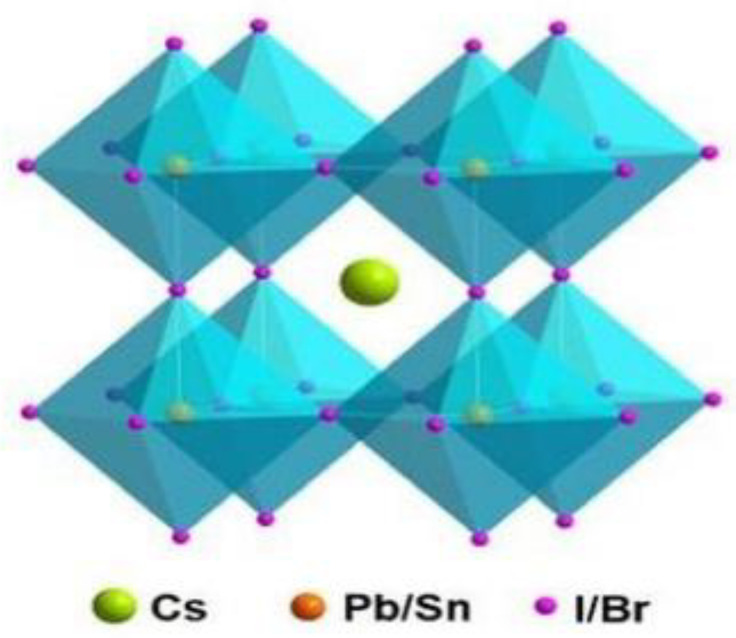
Schematic structure of CsPbX_3_ perovskite [45].

CsPbBr_3_ perovskite material belongs to the orthorhombic crystal system [44], as shown in Figure 5, where Cs^+^ is located in the cell center and forms AX_12_ dodecahedra with neighboring halogen atoms. Pb^2+^ ions at the B-site are coordinated with the neighboring six halogen element ions to form PbX_6_ octahedra. PbX_6_ octahedra extend in three dimensions to form a framework structure.

The Goldschmidt tolerance factor and octahedral factor can be used to measure the stability of the perovskite structure, and the Goldschmidt tolerance factor is as follows:

$$t=\frac{R_{A}+R_{X}}{\sqrt{2}R_{B}+R_{X}}$$

*R_A_*, *R_B_*, and *R*_X_ are the ionic radius of *A*, *B*, *X* sites, respectively. When t = 1, it is an ideal perovskite structure. When t > 1, the structure is a tetragonal crystal system and when t < 1, the perovskite is an octahedral structure [46]. In order to keep the perovskite structure relatively stable, t should satisfy 0.81 < t < 1.11. 

The t value of CsPbBr_3_ is 0.92 [44]. The high t value of CsPbBr_3_ is conducive to the stability of the perovskite phase over a wider temperature range. However, the tolerance factor is not the only measurement of the stability of perovskite materials. Another factor that affects the stability of the perovskite is the octahedral factor, expressed as follows:

$$\mu =\frac{R_{B}}{R_{X}}$$

*R_B_*, *R_X_* is the radius of the B-site and *X*-site ions, respectively. The structure is relatively stable when the *µ* satisfies 0.44 < *µ* < 0.90 [47]. CsPbBr_3_ perovskite exhibits excellent stability under light, heat, and moisture conditions. In general, CsPbBr_3_ has three distinct phases, the orthogonal γ phase, the tetragonal β phase, and the cubic α phase, respectively. At room temperature, CsPbBr_3_ perovskite is the γ phase, which is very stable, but tends to transform into the unstable β and α phases when the temperature reaches 88 °C and 130 °C [44,48]. When the temperature cools to room temperature, the α and β phases return to the γ phase, indicating the excellent stability of CsPbBr_3_ perovskite.

## 3. The Device Structure of PSCs

**Figure 6:**
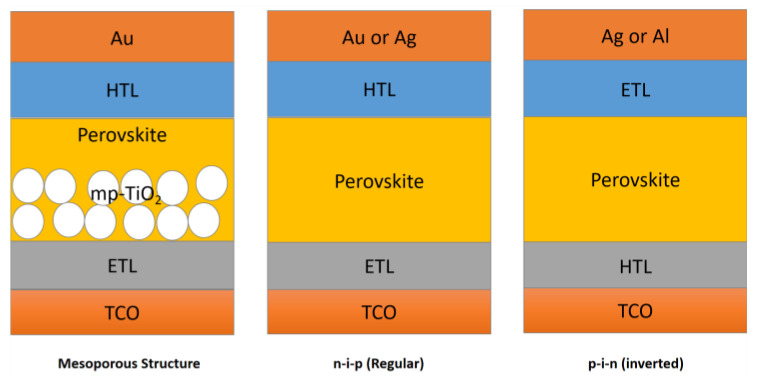
The device structure of PSCs.

The device architecture has a significant impact on the performance of PSCs. The device structure can be mainly divided into two categories: mesoporous structures and planar structures. Planar structures can be divided into the n-i-p (regular) type and the p-i-n (inverted) type, as shown in Figure 6 [49]. Both mesoporous and planar structures consist of an electrode, a carrier transport layer, and a perovskite absorption layer. The perovskite layer is sandwiched between the electron transport layer (ETL) and the hole transport layer (HTL). In mesoporous structure devices, the mesoporous metal oxide skeleton is mainly used as a support in its intermediate pore structure to improve carrier extraction efficiency and the contact area of the interface, thus improving the device performance. However, the manufacturing process of mesoporous cell devices is complicated, and most of them need to be calcined at high temperatures, which increases the device preparation cycle. The manufacturing process of planar cell devices is simple. Furthermore, an interface modification layer can be introduced into the cell structure to optimize interface contact and improve carrier transmission efficiency, resulting in improved device performance.

In PSCs, the most commonly used ETLs are SnO_2_, ZnO, TiO_2_, and phenyl-C61- butyric acid methyl ester (PCBM). The commonly used HTLs are Spiro-OMeTAD (2,2’,7,70-tetrakis(N,N-p-dimethoxy-phenylamino)-9,90-spirobifl-uorene), poly [3hexylthiophene-2,5-diyl] (P3HT), poly triarylamine (PTAA), and NiOx [44]. 

## 4. Mono-Green Solvent Systems

The primary precursors of CsPbBr_3_ in the most commonly used organic solvents are DMF, DMSO, and methanol. Considering the poor solubility of CsBr in methanol, the spin-coating and annealing steps of CsBr/methanol solution should be repeated at least 4–5 times, which not only makes the process complex, but also results in a poor quality of the CsPbBr_3_ film. The volatilization of methanol during the annealing process results in the generation of a large amount of methanol vapor, which can cause serious harm to the biological environment. Therefore, reducing or avoiding the use of methanol and improving the environmental compatibility of CsPbBr_3_-based devices while ensuring device efficiency as much as possible has become an urgent problem.

Water is the most polar solvent in nature. CsBr is very soluble in water at 25 °C (5.8 mol L^−1^) [50]. Extremely little PbBr_2_ dissolves in water (0.026 mol L^−1^) [51]. The enormous solubility gap between PbBr_2_ and CsBr in water makes it possible to prepare CsPbBr_3_ film using a two-step spin-coating method using water as solvent. In 2020, Wei’s group adopted water to dissolve CsBr, and successfully prepared CsPbBr_3_ solar cells with a PCE of 6.12% using a two-step spin-coating method [50]. This method dramatically reduces the cost and processing time, which is beneficial for the development of CsPbBr_3_ PSCs. 

**Figure 7:**
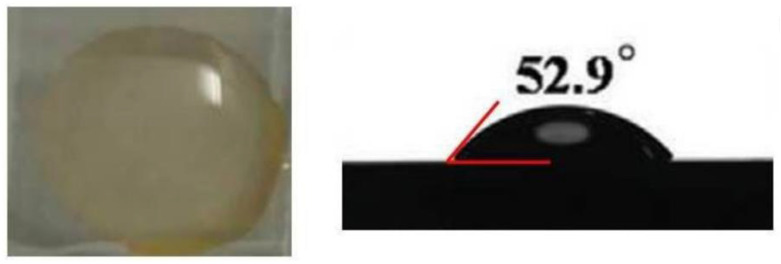
Photo and contact angles of pure PbBr_2_ layer for CsBr/H_2_O solution [52].

However, there are several problems with using water as the solvent for CsBr to prepare CsPbBr_3_ solar cells:

First, the contact property between water and PbBr_2_ films is so poor that CsBr/H_2_O solvent cannot spread uniformly on the surface of PbBr_2_ films, as shown in Figure 7, resulting in inhomogeneous CsPbBr_3_ perovskite film.

Second, water can dissolve the perovskite and destroy the phase of CsPbBr_3_. Meanwhile, the higher boiling point of water results in a slower volatilization rate compared to methanol, which is detrimental to the formation of high-quality CsPbBr_3_ film with high-purity phase.

Third, when using water as the solvent of CsBr, the perovskite films prepared using a two-step spin-coating method will contain many defects due to the high crystallization rate, which will function as nonradiative recombination centers for photogenerated carriers [53].

Finally, using high-concentration CsBr/H_2_O solution can quickly bring about the excess of CsBr, resulting in the penetration of CsBr crystals into the surface of the film. These problems will lead to the bad performance of CsPbBr_3_ solar cells by using water as the solvent of CsBr.

**Figure 8:**
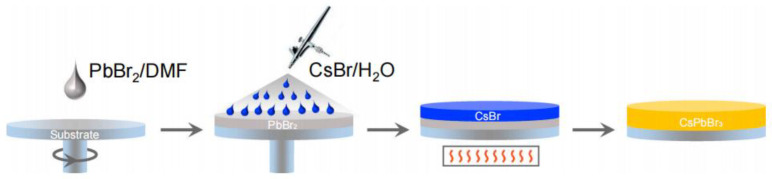
The photo of fabrication of CsPbBr_3_ film by water-based spray-assisted growth strategy [54].

**Figure 9:**
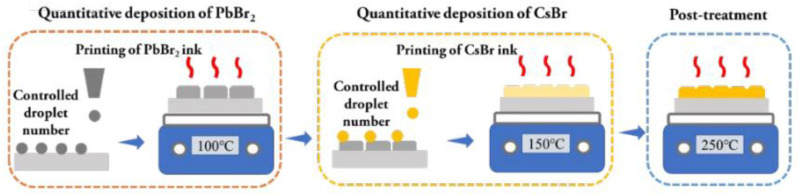
Diagram of the inkjet-printing process of CsPbBr_3_ films [56].

Therefore, how can these problems be solved? Unlike spin-coating methods, one solution is to adopt different fabrication processes for preparing CsPbBr_3_ films. Zhang’s [54] group proposed a water-based spray-assisted growth strategy for CsPbBr_3_ C-PSCs, which involves spraying CsBr aqueous solution onto the deposited PbBr_2_ films, as shown in Figure 8. This strategy effectively solves the problem of poor contact between PbBr_2_ films and water. The champion PCE of the CsPbBr_3_-based solar cells fabricated by the water-based spray-assisted growth strategy reached 10.22%, and the photocurrent output can maintain 90% of its initial value after continuous Test 16 h under simulated AM 1.5 G illumination and in the air with a temperature of 25 °C and relative humidity (RH)of 45%. Xu’s group adopted an inkjet-printing method to prepare CsPbBr_3_ films. Inkjet-printing is to precisely spray droplets with a picoliter volume of perovskite precursor solutions to control the chemical composition of the perovskite films [55]. As shown in Figure 9. First, the PbBr_2_ solution was printed onto the meso-TiO_2_ layer and annealed at 100 °C to accelerate the crystallization process of PbBr_2_. Subsequently, the CsBr solution in a mixed solvent of methanol and water was printed onto the PbBr_2_ layer to form CsPbBr_3_ film. Finally, the CsPbBr_3_ film was post-treated to enhance the quality of printed CsPbBr_3_ film. The inkjet-printed CsPbBr_3_ C-PSCs achieved PCEs of up to 9.09%, 8.59%, and 7.81% with active areas of 0.09 cm^2^, 0.25 cm^2^ and 1 cm^2^ and can maintain 92.8% of initial PCE after thermal stressing at 80 °C for 1650 h, indicating the excellent thermal stability of the inkjet-printed device. [56].

Other solutions include the application of additives, adjusting the orientation and size of grains, regulating the composition of precursor solution, and solvent post-treatment. Ethylene glycol monomethyl ether (EGME, Alias is 2-methoxyethanol) can be used as the solvent of CsBr and can prepare pure-phase CsPbBr_3_ film, but the film morphology is inferior due to the excessive Ostwald ripening [57]. Li’s group [57] proposed a bi-solvent system strategy to adjust the morphology and phase of CsPbBr_3_ films via two-step methods, which adopt the mixed solvent comprising EGME and isopropanol (IPA) in place of EGME for the CsBr solution to prepare smooth, compact, and pure-phase CsPbBr_3_ films. The PCE of the produced cells achieved 7.29%. Chen’s group prepared an Ln_2_S_3_ film for the electron transport layer (ETL) via a facile and controllable reflux condensation technique and used 2-methoxyethanol as the solvent of CsBr to prepare high-quality CsPbBr_3_ PSCs with a PCE of 6.54% [58].

Inspired by the ideas as mentioned above, Wu’s [59] group developed a high-quality CsPbBr_3_ solar cell with a PCE of 9.55% via a triple-step spin-coating method using a bi-solvent system made up of green solvent water and EGME instead of toxic methanol for the CsBr solution. After 34 days of aging, the device PCE in the case of green solution processed cells kept about 86% of its original value, whereas the PCE of the toxic solution-processed devices degraded to 70.3% of its original PCE value, demonstrating the excellent light stability in the green solution processed cells.

Moreover, Zhu’s group [60] discovered that adding a small amount of CsBr additive to the PbBr_2_ solution can promote the reaction of the prepared PbBr_2_ film with the CsBr aqueous solution, which can adjust the crystal orientation of PbBr_2_ from [020] to [031]. A carbon-electrode PSC made using this technique was produced, with a remarkable PCE of 10.27%, and maintained 93.2% of its initial PCE after being stored for 30 days, demonstrating superb stability. This is the highest PCE of green solvent in the CsPbBr_3_ system.

Cao’s group [61] investigated the impact of post-annealing temperature on the performances of the CsPbBr_3_ film prepared by the reaction of CsBr aqueous solution with PbBr_2_ film, which enhanced the device PCE from 2.67% at an annealing temperature of 200 °C to 5.20% at an annealing temperature of 300 °C. Furthermore, a small quantity of reduced graphene oxide (RGO) was doped to mesoporous TiO_2_ (m-TiO_2_), and the prepared CsPbBr_3_ solar cells presented an apparent enhancement PCE of 7.08%. The performance improvement demonstrates that the post-annealing temperature significantly influences the quality of all inorganic perovskite CsPbBr_3_ films.

Sun’s group [62] adopted a facile solution processing method to create high-purity CsPbBr_3_ films. They used the CsBr methanol/H_2_O solution for spin-coating onto the PbBr_2_ film, and then optimized the crystallization and morphology of CsPbBr_3_ films with isopropanol-assisted post-treatment. The perovskite solar cells made in this way with a planar architecture had a PCE of 8.11%. The unencapsulated IPA post-treated device showed excellent humidity stability and thermal stability, maintaining 85% of its initial PCE value under 70 °C for 600 h and 78% of its initial PCE value at 85% RH for 480 h.

The current methanol fabrication process for preparing CsPbBr_3_ PSCs is tedious and time-consuming, because it requires multi-step spin-coating (at least 5–6 times) [63]. Li’s group [63] adopted water to dissolve CsBr to study the link between the phase purity of CsPbBr_3_ and the loading time (t) at which CsBr aqueous solution was deposited to the PbBr_2_ surface. With a loading time of 15 s, the prepared CsPbBr_3_ solar cells yielded a champion PCE of 9.14%. In addition, the PCE only slightly decreased under a humid and high-temperature environment during a 30-day aging test, indicating superior stability.

**Figure 10:**
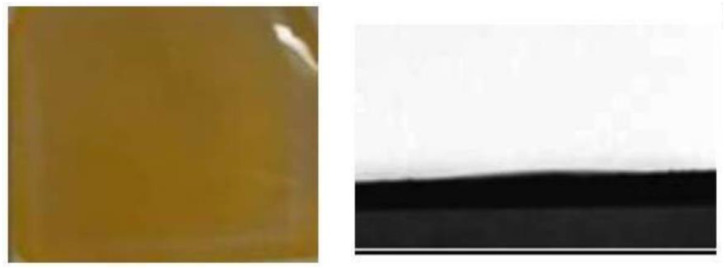
The photo and contact angles of the PbBr_2_/thiourea layer for the CsBr/H_2_O solution [52].

It is essential to boost the wettability of PbBr_2_ films in order to make a high-quality CsPbBr_3_ perovskite film [52]. Wang’s group [52] added thiourea as a functional additive into the PbBr_2_ film in the first step of a two-step spin-coating method, as shown in Figure 10, which essentially promotes the wettability of the PbBr_2_ layer. Thiourea contains the hydrophilic -NH_2_ bond and the C=S bond. Of these, the C=S bond is able to form a Lewis acid–base bond with PbBr_2_. Therefore, the addition of thiourea can significantly improve the hydrophilicity of the PbBr_2_ layer. On the other hand, the thermal decomposition temperature of thiourea is relatively low. When the annealing temperature reaches 250 °C, thiourea will decompose and be removed, which will lead to the appearance of many pores in the PbBr_2_ layer, and some of these pores provide extra space for the growth of perovskite crystals, which is highly favorable for the formation of large crystal grains. Based on this method, the CsPbBr_3_ PSC with an FTO/TiO_2_/CsPbBr_3_/carbon structure exhibits superior performance with a high efficiency of 9.11%. After 45 days of storage, the CsPbBr_3_ PSC was stable, still maintaining over 90% of its original efficiency.

He’s group [53] added a self-polymeric monomer of N-(hydroxymethyl) acrylamide (HAM) containing C=C, C=O, and -NH multifunctional groups in CsBr aqueous solution to prepare CsPbBr_3_ PSCs using a two-step method. The best power conversion efficiency of the cell was 9.05%. Adding HAM additive to CsBr aqueous solution to prepare CsPbBr_3_ cells was able to not only retard the crystallization, but also to regulate the band structure of the CsPbBr_3_ layer and simultaneously passivate its defects. Moreover, the device maintained over 92% of its initial efficiency value after 30 days of storage in ambient air under highly humid conditions or at high temperature, demonstrating significant stability. 

## 5. All-Green Solvent Systems

In the above-mentioned research ideas, PbBr_2_ films were first spin-coated onto substrates from PbBr_2_/DMF solution, and then the CsBr green solution was spin-coated on the prepared PbBr_2_ layer to create CsPbBr_3_ film.

It appears that our only option is to adopt toxic DMF in the first step during the preparation of perovskite films due to the low solubility of PbBr_2_ in green solvent. However, if the human body is exposed to an environment containing high levels of DMF, it can cause a lot of harm, especially to the human liver [32]. Thus, it is necessary to develop an all-green solvent system to prepare CsPbBr_3_ PSCs to guarantee the health of humans. 

**Figure 11:**
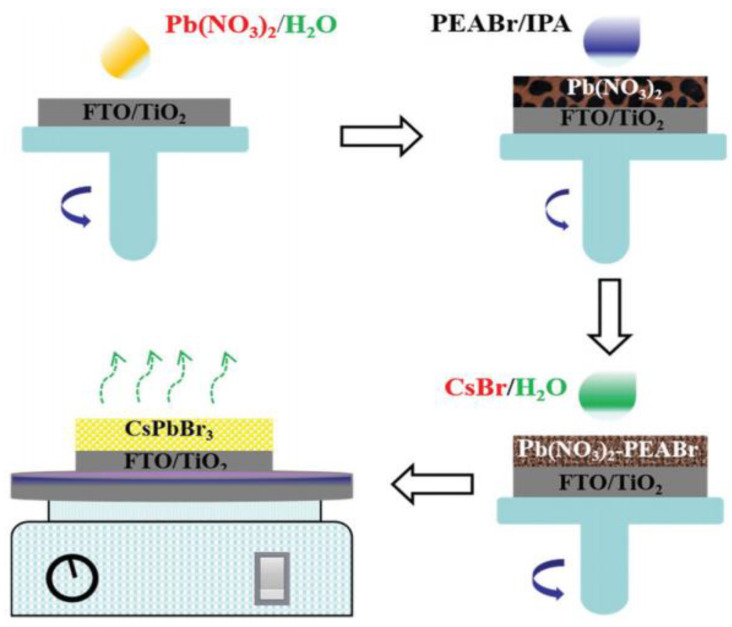
Schematic diagram of the eco-friendly fabrication process of CsPbBr_3_ films [64].

Generally, there are two ways to prepare CsPbBr_3_ PSCs in all-green solvent systems. The first method is to prepare PbBr_2_ films with a two-step spin-coating method, followed by reacting with CsBr aqueous solution. Wei’s [64] group created an innovative method to realize the eco-friendly manufacture of CsPbBr_3_ films using water to dissolve precursors of Pb(NO_3_)_2_ and CsBr, and using IPA to dissolve phenethylammonium Bromide (PEABr), as shown in Figure 11. In this method, only the two green solvents of water and 2-propanol (IPA) were used to prepare CsPbBr_3_ PSCs with a champion PCE of 6.25%. When kept at room temperature for 30 days with a relative humidity of 40%, the device maintains 94% of initial PCE, showing the superb stability of CsPbBr_3_ cells. This work described good previous experience for the eco-friendly manufacture of CsPbBr_3_ PSCs, and reduces the manufacturing cost of the CsPbBr_3_ PSCs. Furthermore, this innovative method for preparing CsPbBr_3_ film is entirely different from the frequently used multi-step spin-coating method, and is very instructive for the preparation of CsPbBr_3_ PSCs. However, the PCE of CsPbBr_3_ solar cells prepared by this method is relatively low.

Another method is to find a green or green-mixed solvent to replace DMF to dissolve PbBr_2_. Wei’s group [65] adopted all-green solvents based on a two-step spin-coating method to make CsPbBr_3_ films, in which a green mixed solvent of γ-butyrolactone and polyethylene glycol (PEG) is used to dissolve PbBr_2_ in the first step and green water to dissolve CsBr to prepare CsPbBr_3_ PSCs with a PCE of 8.11%. The highest PCE of the all-green solvent system of CsPbBr_3_ PSCs, in which polyethylene glycol played a solubilizing effect, was 8.11%. After 30 days of air storage, the device’s PCE still exhibited performance similar to that of the original value, demonstrating the excellent stability of CsPbBr_3_-based solar cells.

**Figure 12:**
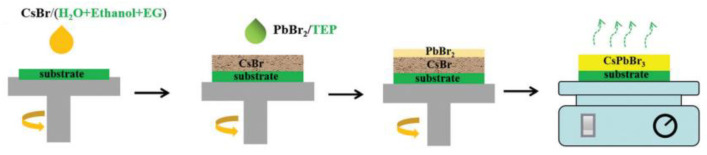
Schematic image of the fabrication process of CsPbBr_3_ films through an anomalous route using all-green solvents [32].

Generally speaking, PbBr_2_ has low solubility in commonly used green solvents. The solubility of PbBr_2_ in triethyl phosphate (TEP) is 60 mg/mL [32], which is much lower than the solubility of PbBr_2_ in DMF. Thus, Wei’s [32] group developed an anomalous sequential deposition method to make CsPbBr_3_ films, as shown in Figure 12, in which the CsBr solution is prepared using a green solvent system made of water, suitable ethanol, and ethylene glycol (EG), and the PbBr_2_ solution by using a green TEP. In contrast to the conventional sequential deposition method, CsBr solution is first deposited in the first step, and then reacted with PbBr_2_. In this anomalous sequential deposition method, the cells exhibit a PCE of 6.86%, which is equivalent to the PSCs prepared using toxic solvents. Moreover, the PCE of the device shows a comparable value to the initial value when the device was kept in air for 14 days with 40% RH and at 30 °C, displaying the outstanding stability of CsPbBr_3_ solar cells.

**Figure 13:**
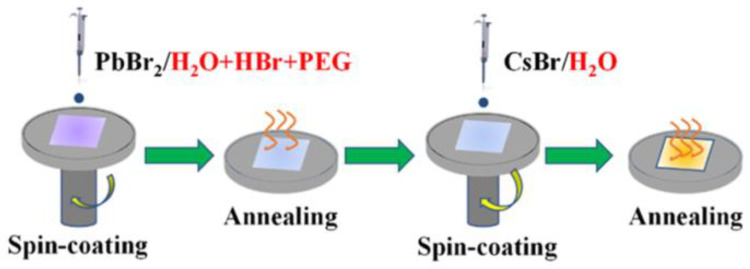
Schematic diagram of the fabrication process of CsPbBr_3_ films from aqueous solutions [66].

Water is the most abundant substance in nature. If we use water to replace DMF as the solvent of PbBr_2_, it will primarily reduce the cost of CsPbBr_3_ PSCs and benefit the environment. Wei’s group adopted all-aqueous solutions to prepare all-inorganic CsPbBr_3_ perovskite films, in which the HBr/H_2_O solution containing a small amount of poly (ethylene glycol) (PEG) was used to dissolve PbBr_2_ and H_2_O to dissolve CsBr, as shown in Figure 13. The CsPbBr_3_ PSCs prepared in this way show a PCE of 7.19%, and the PCE of PSC maintains a maximum PCE of 95.8% after 22 days [66]. These efforts make it possible to fabricate perovskite films using all aqueous solutions.

## 6. Approaches to Improving PCE in CsPbBr_3_ Perovskite Cells

Compared with the preparation of CsPbBr_3_ cells via traditional toxic solvent methods, the use of green solvent in the preparation of CsPbBr_3_ cells reduces the PCE of the devices. What can be done to improve the PCE of the perovskite cells reduced by green solvent method?

A good approach is the metallization of PSCs. As is well known, metallic nanoparticles can increase perovskite cell efficiency owing to the plasmon mediated photovoltaic effect. The microscopic theory of plasmon-mediated photovoltaic effect includes the optical and electrical channels of plasmon mediation [67]. Through the optical channel of plasmon mediated photovoltaic effect, the photo efficiency of solar cells will be greatly improved. The enhancement of photo efficiency of solar cells through metallic nanoparticles in the optical channel of plasmon-mediated photovoltaic effect occurs due to a strong vibration of surface plasmons in metallic nanoparticles, which overlaps with the visible sunlight spectrum and causes highly efficient absorption of the incident photo. In the electrical channel of the plasmon-mediated photovoltaic effect, the reduction in binding energy occurs as a result of metallic nanoparticles, thus increasing the efficiency, which is dominant in perovskite cells. Janusz E. Jacak’s group included Au/SiO_2_ nanoparticles in the Al_2_O_3_ or TiO_2_ basis of CH_3_NH_3_PbI_3−*α*_Cl*_α_* hybrid perovskite cells, the application of which enhanced the PCE from 10.7% to 11.4% and from 8.4% to 9.5%, mainly owing to the previously mentioned plasmon-mediated photovoltaic effect [68]. Furthermore, Witold Aleksander Jacak’s group demonstrated that using multi-shell elongated metallic nanoparticles in a perovskite structure can boost the photo absorption (related to the optical plasmon photovoltaic effect) while decreasing the binding energy of excitons (related to the electric plasmon photovoltaic effect), thereby improving the PCE [69]. At present, there has been little research on the metallization of CsPbBr_3_ PSCs via green solvents. We think this direction is worth studying.

Of course, some other approaches exist for increasing the PCE of low-efficiency CsPbBr_3_ PSCs. The approaches to increasing the efficiency of PSCs are summarized as anti-solvent engineering, the application of additives, structure engineering of grain orientation and size, the modification of the precursor solution, interface modification, and innovative post-treatment [70]. In particular, increasing the grain size of CsPbBr_3_ perovskite crystals, reducing passivation defects, and improving carrier transport and separation are promising for greatly improving the PCE of the device. The previous discussion explored the application of additives, structure engineering of grain orientation and size, innovative post-treatment, and the modification of the precursor solution. However, compared with traditional preparation methods via toxic solvents, these modified approaches still need to be applied in the preparation method via green solvents. Currently, in the development of green solvents in CsPbBr_3_ PSCs, anti-solvent engineering and interface modification has yet to be explored. Future researchers can explore the above research ideas.

## 7. Conclusions and Prospects

The use of toxic solvents in the preparation of CsPbBr_3_ PSCs can greatly hinder their commercial application. Replacing toxic solvents with green solvents that are beneficial to the environment and the health of human beings is a vital step before CsPbBr_3_ perovskite solar cells can move to market. In this paper, we introduced the crystal structure and basic properties of CsPbBr_3_ and the device structure of PSCs, summarized the research progress of green solvent in CsPbBr_3_ perovskite solar cells from mono-green solvent systems to all-green solvent systems. This dramatically reduces the toxicity in the preparation of CsPbBr_3_ perovskite solar cells. Furthermore, we also discussed the approaches to improving the PCE of CsPbBr_3_ perovskite cells, the metallization of perovskite, anti-solvent engineering, the application of additives, structure engineering of grain orientation and size, the modification of the precursor solution, interface modification and solvent post-treatment approaches, all of which are well worth exploring. The performance of the CsPbBr_3_ PSCs prepared by the green solvent is shown in Table 1 and Table 2.

For mono-green solvent systems, water or mixed solvents containing water (such as H_2_O/methanol, Ethylene glycol monomethyl ether/H_2_O) are generally used to dissolve Cesium Bromide due to the high solubility of water for CsBr, and the low solubility of methanol for CsBr. Ethylene glycol monomethyl ether (EMGE), used as a solvent for CsBr to prepare CsPbBr_3_ film, can effectively inhibit the appearance of CsPb_2_Br_5_ and Cs_4_PbBr_6_ phase, and the specific intrinsic mechanism is worthy of exploration by subsequent researchers. The CsPbBr_3_ film prepared by mixed solvents containing EGME (e.g., EGME/IPA, EGME/H_2_O) to dissolve CsBr has higher purity in the CsPbBr_3_ phase. Water-based spray-assisted growth methods and inkjet printing methods are more accessible for the preparation of high-purity and uniform CsPbBr_3_ perovskite films than multi-step spin-coating methods. PbBr_2_ films have poor wettability, and the addition of thiourea to PbBr_2_ can very effectively improve the wettability of PbBr_2_ films and promote the reaction of PbBr_2_ films with CsBr aqueous solutions. Meanwhile, adding N-(hydroxymethyl) acrylamide (HAM) to the CsBr aqueous solution leads to a better crystalline quality of the grown CsPbBr_3_ films, because, with the change in temperature, HAM undergoes a self-polymerization reaction that slows down the crystallization rate of CsPbBr_3_. Among them, the maximum PCE of CsPbBr_3_ solar cells with mono-green solvent system was 10.27%, which is comparable to the CsPbBr_3_ PSCs prepared by toxic solvents. The selection of water as the solvent dramatically reduces the cost and pollution of the preparation process, which is very significant for the commercialization and sustainable development of CsPbBr_3_ perovskite solar cells. It may be common to use green solvents to prepare CsPbBr_3_ PSCs in the future.

For all-green solvent systems, water or a mixed green solvent are generally used to dissolve CsBr, and some green solvents are selected to replace DMF. To date, there are still relatively few studies on all-green solvents of CsPbBr_3_, and the PCE of the CsPbBr_3_ PSCs prepared using all-green solvent systems is relatively low, with the highest power conversion efficiency being only 8.11%. DMF is a significant hazard to the human body and the environment, while the PCE of CsPbBr_3_-based PSCs prepared by all-green solvents is generally low (lower than 9%). Therefore, it is still very promising and significant to continue exploring all-green solvent systems to prepare outstandingly efficient CsPbBr_3_ PSCs.

## Figures and Tables

**Table 1:** Recent progress in mono-green solvent-based CsPbBr_3_ PSCs photovoltaic performance.

Solvent or Additive for PbBr_2_	Solvent or Additive for CsBr	Fabrication Method	Jsc (mA·cm^−^^2^)	Voc (V)	PCE(%)	FF	Ref.
DMF	H_2_O	Spin-coating method	7.48	0.688	6.12	0.688	[50]
DMF + DMSO(9:1)	Methanol + H_2_O(5:1)	Spin-coating method	6.89	1.49	8.11	0.79	[62]
DMF + DMSO(1:1)	H_2_O	Spin-coating method	7.96	1.555	10.27	0.83	[60]
DMF	2-methoxyethanol	Spin-coating method	6.96	1.36	6.54	0.69	[58]
DMF	Methanol + H_2_O(6:1)	Spin-coating method	7.28	1.28	7.08	0.76	[61]
DMF	EGME/IPA	Spin-coating method	7.12	1.49	7.29	0.688	[57]
DMF	H_2_O	Spin-coating method	7.64	1.52	9.14	0.79	[63]
DMF	H_2_O	Water-based spray-assisted growth method	8.06	1.528	10.22	0.83	[54]
DMF + DMSO(9: 1)	Methanol:H_2_O(10: 1)	Inkjet-printing method	7.36	1.512	9.09	0.817	[56]
DMF	H_2_O + EGME(1:1)	Spin-coating method	7.48	1.51	9.55	0.844	[59]
DMF + Thiourea	H_2_O	Spin-coating method	8.81	1.38	9.11	0.75	[52]
DMF	H_2_O +HAM	Spin-coating method	7.54	1.468	9.05	81.76	[53]

**Table 2:** Recent progress in all-green solvent-based CsPbBr_3_ PSCs photovoltaic performance.

Solvent or Additive for PbBr_2_	Solvent or Additive for CsBr	Fabrication Method	Jsc (mA·cm^−^^2^)	Voc (V)	PCE(%)	FF	Ref.
TEP	H_2_O:EA:ethylene glycol(1:1:0.1)	Anomalous sequential Spin-coating method	7.04	1.30	6.86	0.75	[32]
H_2_O + HBr + PEG	H_2_O	Spin-coating method	7.18	1.32	7.19	0.759	[66]
γ-butyrolactone + PEG	H_2_O	Spin-coating method	7.78	1.22	6.89	0.725	[65]
Pb(NO_3_)_2_/H_2_O + PEABr/IPA	H_2_O	Spin-coating method	6.12	1.44	6.25	0.709	[64]

## Data Availability

The systematic review data used to support the findings of this study are included with the article.
